# Supplementary material for: The Representation of Orientation Semantics in Visual Sensory Memory
Source: Behav Sci (Basel). 2024 Dec 24;15(1):1. doi: 10.3390/bs15010001 (PMC11759768; doi:10.3390/bs15010001)
Supplement: Supplementary file 1 [file behavsci-15-00001-s001.zip › behavsci-3309377-supplementary.pdf]

Supplemental Materials

Analysis for Response Times (RTs) in all Experiments

A 2 (mask type) × 3(report type) repeated measures ANOVA was conducted. In Experiment 1, no significant main effect of mask type was found ($F(1, 23) = 1.93$, $p = .178$, $\eta_p^2 = .077$). The main effect of report type was significant ($F(2, 46) = 52.25$, $p < .001$, $\eta_p^2 = .694$), but the interaction effect was not significant ($F(2, 46) = 1.18$, $p = .317$, $\eta_p^2 = .049$). Further comparison showed that in all report types, the RTs were not different significantly (with Bonferroni correction: $p > .500$ for partial report with cue deley 0ms; $p = .105$ for partial report with cue deley 447ms; $p = .242$ for whole report).

In Experiment 2, no significant main effect of mask type was found ($F(1, 23) = 0.03$, $p > .500$, $\eta_p^2 = .001$). The main effect of report type was significant ($F(2, 46) = 64.85$, $p < .001$, $\eta_p^2 = .738$), but the interaction effect was not significant ($F(2, 46) = 0.27$, $p > .500$, $\eta_p^2 = .012$). Further comparison showed that in all report types, the RTs were not different significantly (with Bonferroni correction: $p > .500$ for all three report types).

In Experiment 3, no significant main effect of mask type was found ($F(1, 23) = 0.10$, $p > .500$, $\eta_p^2 = .004$). The main effect of report type was significant ($F(2, 46) = 50.78$, $p < .001$, $\eta_p^2 = .688$), but the interaction effect was not significant ($F(2, 46) = 0.21$, $p > .500$, $\eta_p^2 = .009$). Further comparison showed that in all report types, the RTs were not different significantly (with Bonferroni correction: $p > .500$ partial report with cue deley 0ms and whole report; $p = .443$ for partial report with cue deley 447ms).

Table S1. Response Times (RTs) in all three experiments

		Cue delay 0ms	Cue delay 447ms	Whole report
**Exp. 1**	**Circle**	4409ms (1458)	4348ms (2121)	11978ms (4627)
	**Triangle**	4402ms (1327)	4834ms (2256)	14296ms (9350)
**Exp. 2**	**Square**	4658ms (1762)	4618ms (1374)	15633ms (7985)
	**Rectangle**	4936ms (2589)	4727ms (2021)	14728ms (9770)
**Exp. 3**	**Chinese character without orientation**	4140ms (1783)	3881ms (1448)	10425ms (5955)
	**Chinese character with orientation**	4365ms (1772)	3727ms (1141)	10842ms (6415)

Note: The values in brackets represent standard deviations.

Analysis for target-mask pairs in Experiment 3

We compared whether the target-mask congruent condition will be different from target-mask incongruent. The target-mask congruent means the target had the same direction with the mask (e.g., a vertically upward arrow masked by the Chinese character "up"), and the target-mask incongruent means all other situations. Since some positions in each trial may be task-mask congruent while others are incongruent, we calculated the number of congruent and

incongruent items for each trial, as well as the number of correct reports for these items. Finally, the average accuracy of each participant in both the congruent and incongruent conditions was calculated and multiplied by the total number of items (12) to determine the number of correct recalls. A 2 (target-mask congruency) × 3(report type) repeated measures ANOVA was conducted. Significant main effect of target-mask congruency was found (F(1, 23) = 6.77, p = .016, η_p^2 = .227). Neither the main effect of report type (F(2, 46) = 0.03, p > .500, η_p^2 = .001), nor the interaction effect was significant (F(2, 46) = 1.54, p = .225, η_p^2 = .063). Further comparison showed that the number of correct reports in congruent condition were significantly higher in cue delay 447ms (with Bonferroni correction: p = .081 for partial report with cue deley 0ms; p = .013 for partial report with cue deley 447ms; p > .500 in whole report).

Table S2. Number of correct reports in Experiment 3

	**Cue delay 0ms**	**Cue delay 447ms**	**Whole report**
**Target-mask congruent**	2.87 (0.26)	2.96 (0.27)	2.65 (0.25)
**Target-mask incongruent**	2.40 (0.14)	2.23 (0.14)	2.53 (0.13)

Note: The values in brackets represent standard deviations.

Table S3. The effect of mask orientation relative to target orientation in Experiment 3

		**Correct recall number**	***P* value**				
			**45°**	**90°**	**135°**	**180°**	**Without orientation**
**Cue delay 0ms**	**0°**	2.87 (0.26)	> .500	> .500	.171	> .500	> .500
	**45°**	2.26 (0.18)	-	> .500	> .500	> .500	.379
	**90°**	2.56 (0.17)	-	-	.249	> .500	> .500
	**135°**	2.09 (0.20)	-	-	-	.046*	.004*
	**180°**	2.88 (0.25)	-	-	-	-	> .500
**Cue delay 447ms**	**0°**	2.96 (0.27)	.085	> .500	.103	> .500	> .500
	**45°**	1.96 (0.16)	-	.021*	> .500	.515	.001*
	**90°**	2.58 (0.19)	-	-	.041*	> .500	> .500
	**135°**	1.99 (0.16)	-	-	-	.410	< .001*
	**180°**	2.52 (0.23)	-	-	-	-	> .500
**Whole report**	**0°**	2.65 (0.25)	> .500	> .500	> .500	> .500	> .500
	**45°**	2.39 (0.14)	-	> .500	> .500	.178	.064
	**90°**	2.67 (0.20)	-	-	.740	> .500	> .500
	**135°**	2.27 (0.15)	-	-	-	.203	.025*
	**180°**	2.97 (0.26)	-	-	-	-	> .500

Note: The values in brackets represent standard deviations. The asterisks (*) indicate p < .050

The data were further categorized into six levels based on the difference in orientation between the mask and the target: 0°, 45°, 90°, 135°, 180°, and Chinese characters without orientation. The results demonstrated that the orientation difference between the mask and the target had a significant main effect (F(5, 115) = 7.92, p < .001, η_p^2 = .256). The masking effect was weakest when the mask and the target shared the same orientation (0° condition), with the correct recall number in this condition (2.83) being even higher than in the condition without

an orientational mask (2.72). In contrast, the masking effect was strongest when the orientation difference was 45° or 135°, with the correct recall number significantly lower than in the condition without an orientational mask. This pattern of results remained consistent across different report types (see Table S3).

Analysis for effect of target orientation in Experiment 3

We compared whether the vertical and horizontal target were recalled better than diagonal target in Experiment 3. Only data in the condition using Chinese characters without orientational information were included. Both the main effect of target orientation ($F(1, 23) = 19.03$, $p < .001$, $\eta_p^2 = .453$) and its interaction with report type were significant ($F(2, 46) = 3.17$, $p = .051$, $\eta_p^2 = .121$). Further comparsion revealed that vertical/horizontal target were recalled better than diagonal target in both cue delay 0ms and cue delay 447ms condition (see Table S4).

Table S4. Correct recall number for vertical/horizontal versus diagonal target

**Exp. 3 Chinese character without orientation**	**Vertical/horizontal target**	**Diagonal target**	***P* value**
**Cue delay 0ms**	2.92 (0.15)	2.49 (0.15)	.003*
**Cue delay 447ms**	3.03 (0.19)	2.24 (0.12)	.001*
**Whole report**	2.94 (0.14)	2.70 (0.12)	.167

Note: The values in brackets represent standard deviations. The asterisks (*) indicate p < .050

We conducted a separate analysis of the condition where the targets were diagonal, and the results consistently demonstrated a stronger masking effect when the mask consisted of Chinese orientation characters ($F(1, 23) = 14.83$, $p = .001$, $\eta_p^2 = .392$). The main effect of report type also reached significance ($F(2, 46) = 5.42$, $p = .008$, $\eta_p^2 = .191$), while the interaction effect was not significant ($F(2, 46) = 0.21$, $p > .500$, $\eta_p^2 = .009$). Further analysis showed that, across all three report types, the correct recall number under the orientational mask condition was significantly lower than under the non-orientational mask condition (with Bonferroni correction: $p = .034$ for partial report with cue deley 0ms; $p = .013$ for partial report with cue deley 447ms; $p = .009$ for whole report). The correct recall number in different report type could be seen in Table S5. These findings further support the conclusion that when the mask and target orientations differ (as in Experiments 1 and 2), the semantic information conveyed by the mask's orientation creates conflict and interferes with target memory.

Table S5. Correct recall number for diagonal target

Mask type	Report type	Correct recall number	*P* value	
			Cue delay 447ms	Whole report
With orientation	Cue delay 0ms	2.19 (0.16)	.392	> .500
	Cue delay 447ms	1.98 (0.15)	-	.062
	Whole report	2.33 (0.11)	-	-
Without orientation	Cue delay 0ms	2.49 (0.15)	.308	> .500
	Cue delay 447ms	2.24 (0.12)	-	.006*
	Whole report	2.70 (0.12)	-	-

Note: The values in brackets represent standard deviations. The asterisks (*) indicate p < .050

Analysis focued only on the targets were vertical and horizontal also showed a stronger masking effect when the mask consisted of Chinese orientation characters ($F(1, 23) = 5.16$, $p = .033$, $\eta_p^2 = .183$). The main effect of report type ($F(2, 46) = 0.07$, $p > .500$, $\eta_p^2 = .003$) and the interaction effect was not significant ($F(2, 46) = 0.61$, $p > .500$, $\eta_p^2 = .026$). Further analysis with Bonferroni correction showed that the correct recall number under the orientational mask condition was significantly lower than under the non-orientational mask condition ($p = .023$) for partial report with cue deley 447ms ($p = .259$ for partial report with cue deley 0ms; $p = .322$ for whole report).
